# “Teach for ethics in palliative care”: a mixed-method evaluation of a medical ethics training programme

**DOI:** 10.1186/s12904-020-00653-7

**Published:** 2020-09-25

**Authors:** Ludovica De Panfilis, Silvia Tanzi, Marta Perin, Elena Turola, Giovanna Artioli

**Affiliations:** 1 Unit of Bioethics, Azienda USL-IRCCS di Reggio Emilia, Reggio Emilia, Italy; 2 Palliative Care Unit, Azienda USL-IRCCS di Reggio Emilia, Reggio Emilia, Italy; 3 PhD Program in Clinical and Experimental Medicine, University of Modena and Reggio Emilia, Modena, Italy; 4 Scientific Directorate, Azienda USL-IRCCS di Reggio Emilia, Reggio Emilia, Italy

**Keywords:** Ethics, Palliative care, Education, Ethical analyses, Program evaluation

## Abstract

**Background:**

Training in medical ethics aims to educate health care professionals in dealing with daily care ethical issues. To guarantee quality of life and spiritual and emotional support, palliative care professionals have to develop ethical and relational skills. We propose the implementation and evaluation of a specialized training programme in medical ethics dedicated to a hospital-based Palliative Care Unit.

**Methods:**

This study is a mixed-method before-after evaluation with data triangulation.

**Results:**

The results highlight that participants developed their ethical knowledge, and a deeper ethical awareness. They also felt more confident and motivated to widely apply ethical reflections and reasonings in their daily practice.

**Conclusion:**

The participants appreciated the innovative structure of the training, especially regarding the integration of the theoretical-interactive and practical parts. However, they recommended increasing the number of concrete occasions for ethical supervision and practical application of what they learned during the programme. The training programme also has some potential practical implications: the development of advanced ethical skills within a hospital-based PC team may improve the quality of life of the patients and their families. In addition, health care professionals with advanced ethical competencies are able to educate patients and their families towards more active participation in the decision-making process.

## Background

Medical ethics can be defined in several ways, depending on the selection of a normative approach or an empirical one. For the purpose of this paper, we can define medical ethics as more than an academic discipline. According to Farsides “rather medical ethics is a form of practice directed towards identifying and addressing problems which are rarely if ever purely theoretical or predominantly conceptual” [1].

Training in medical ethics aims to educate health care professionals in dealing with daily care ethical issues [2, 3]. Training can take a theoretical approach to medical ethics, based on the idea that moral skills can arise through conceptual theories, and in doing so, they aim to develop the human side of health care professionals [4]. Alternatively, an empirical approach to teaching medical ethics aims at providing the basis for developing ethical skills, such as how to conduct moral reasoning, how to perform an ethical analysis, or how to manage ethical dilemmas [5].

Palliative care professionals often have to deal with ethical dilemmas and moral distress about the best thing to do [6]. Due to the characteristics of the patients involved in the palliative care setting, the severity of the disease and the end of life discussion, and the most recent definition of the discipline [7], palliative care professionals must develop not only clinical, communication and relational skills but also ethical ones. Ethical skills include the management of ethical dilemmas and the process of shared decision making based on the patients’ values and preferences in order to guarantee quality of life and spiritual and emotional support.

While developing the training programme, we reviewed the literature to investigate the methods and strategies being applied in ethics educational programmes. According to our review, ethical training in the palliative care field has increased over the last ten years, especially in South America and Spain. The students involved are primarily medical and nursing students [8–11]. There are few experiences described in the literature involving other health care professionals [5, 12, 13]. Moreover, the training programmes for already qualified doctors or nurses use different educational methodologies, and there is no apparent consistency with regard to methods, content, or length. Last, the training programmes have been evaluated in terms of the participants’ perception and satisfaction before and after the program [8–13].

We propose the implementation and evaluation of a specialized training programme in medical ethics dedicated to a hospital-based Palliative Care Unit (PCU).

The MRC framework for developing and evaluating complex interventions is the methodological framework selected for the project. The MRC framework has a phased approach, from a pre-clinical research phase to a final phase in which the intervention is introduced into the health service: a “bottom up” development which guarantees to enter a phase III trial with an appropriate theory and pilot work [14].

According to this framework, this is a phase 1 study of Feasibility/Piloting. Both quantitative and qualitative methods can be used and integrated [14].

The Moore model is the evaluation framework of this study project with five orders of learning, from Attendance (Level 1) to Change in practice performance (Level 5) [15]. The model is usually used in the continuing medical education programme (CME).

The study aimed to evaluate, both quantitatively and qualitatively, the impact of the training related to:

- Increasing of ethical skills in simulation scenarios (Moore Level 3);
- Evaluation of ethical skills in terms of participant’s competencies and performance (Moore Levels 4 and 5).

## Methods

This study is a mixed-method before-after evaluation with data triangulation. It consists of qualitative and quantitative data collection in different periods (T0,T1,T2); a subsequent separate analysis; and, finally, a comparison of the results (Table 1). The Moore model includes subjective as well as objective evaluation. We performed a subjective self-reported assessment on level 3B: “learning knowledge” to be understood as the degree to which participants *state how* to do what the CME activity intended them to know how to do; on level 4: competence, that is the degree to which participants *show how* to do what the CME activity intended them to be able to do in an educational setting; on level 5: Performance, that is the degree to which participants do what the CME activity intended them to be able to do in their practices.

**Table 1:**
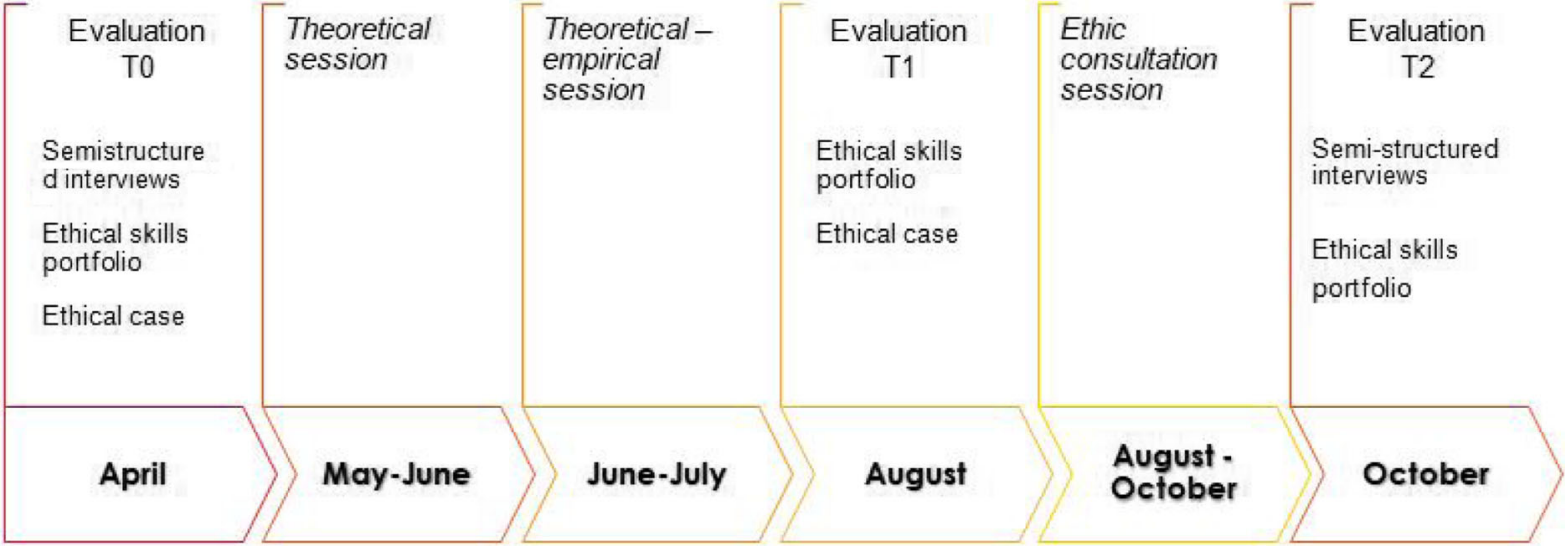
Description of the training program’s structure and evaluation

### Study context

The study was performed in Italy at the oncological research public hospital “Arcispedale Santa Maria Nuova” of the local health authority of Reggio Emilia. It was promoted by the Bioethics Unit (BU) of the hospital and dedicated to health care professionals working at the hospital-based PCU. The purpose of the BU is to assess and promote quality of care for patients, family caregivers and health care professionals throughout empirical bioethics research projects, educational programmes and training programmes, and ethics consultation activities. The projects are focused on developing, implementing, and evaluating new and innovative bioethical interventions at the hospital and community level. These interventions are dealing with ethical issues (i.e., informed consent, shared decision making, end-of-life issues, paediatric issues, patient engagement, truth telling, decision-making capacity, and healthcare costs) in clinical practice and they integrate empirical analysis into ethical theorising. The hospital-based PCU has three main goals: providing specialist consultations for patients (and patients’ family members) hospitalized in the hospital wards or attending the outpatient clinic, promoting research activities, and conducting educational programmes with health care professionals on PC issues. The hospital-based PCU and BU activity started in 2013 and 2016, respectively.

### The intervention

The training programme has been called *Teach for Ethics in Palliative Care (T4EPC).* It involves 28 h of training conducted over 36 weeks. The training programme was held at the hospital where the participants work.

The training focuses on a theoretical session (8 h in three meetings); a theoretical-empirical session (10 h in three meetings); and a session focused on individual ethics consultation on demand (10 h in 5 meetings).

The theoretical session provided knowledge about national and international ethical guidelines and national rules, biolaw issues, normative ethics, advance care planning, and ethics consultation theories. This session involves four external teachers with different educational backgrounds. They are one philosopher, two bioethicists, and one legal expert.

The theoretical-empirical session developed health care professionals’ abilities in conducting ethical analyses, and resolving moral conflicts and ethical dilemmas. This session focused on conducting case-based discussions and supervising the weekly meetings of the hospital-based PCU to explore possible participant’s ethical dilemmas arose in clinical practice.

The ethics consultation session analysed ethical issues that arise in the participants’ daily activities of care. They are real-life consultations or reflection on real life cases at the request of participants.

The PI of this study, the head of the BU of the hospital, conducted the theoretical-empirical session and the ethics consultations.

### Sample and participant recruitment

Participants, including physicians, nurses, and psycho-oncologists working with patients with palliative care needs, were recruited from the hospital-based PCU. The setting was selected purposefully based on the hospital-based PCU characteristics and composition. All interested participants were accepted to attend the training programme.

### Data collection

Data were collected through semi-structured interviews, an ethical skills portfolio and an ethical case analysis.

#### Semi-structured interview

Two researchers on the team with experience in qualitative methods (GA, MP) conducted the interviews following the guide prepared by the research team. The interview covered three thematic areas developed by the PI of the study (LDP):

A) Participant’s expectations;
B) Ethical issues of their clinical practice;
C) Professional needs

The interviews were performed before the training (T0) and at the end of the ethics consultations (T2).

Interviews performed before the training (T0) were also used to revise the content of the course on the participant’s needs. The interview guide is described in Appendix A.

#### Ethical skills portfolio

To carry out the self-reported evaluation of knowledge, competence and performance acquired during the training, we developed a portfolio consistent with the Levati and Saraò model [16].

The developed portfolio is divided into 6 sections designed to described knowledge (section i and ii); competence (section iii and iv); and ethical behaviours (section v and vi). In particular they covered the following ethical issues: i. knowledge of the main ethical and legal sources; ii. knowledge of advanced care planning; iii. Development of critical thinking; iv. ethical analysis of cases; v. development of ethical behaviour; and vi. conduction of ethical consultations. Each of these sections consists of 1 to 7 items that has been defined according to the EAPC core competency [17] and the Royal College of physician training board curriculum [18]. The developed portfolio allowed participants to track, record and reflect on real life experiences or simulated experiences before and after the training.

Data were collected through a self-evaluation scale from poor to excellent for each statement.

It was presented and administered to participants by a researcher (GA) with experience in the evaluation of training programmes.

Participants were asked to self-reported the evaluation of knowledge, competence and performance before the training (T0), at the end of the theoretical part (T1), and at the end of the ethics consultations (T2). At the end of the ethics consultations (T2), participants were asked to complete only sections v and vi.

#### Ethical case analysis

At the beginning (T0) and at the end of the theoretical part (T1) of the training programme, we administered an ethical case [19] that focused on an ethical dilemma in clinical practice (GA,MP). Participants were asked to complete an open-ended question related to this case. The ethical case and the open-ended questions are described in Table 2.

**Table 2:** Ethical case description and related open-ended question

Case Report	Middle aged male with advanced cancer arrived in the Emergency Department (ED) with respiratory distress requiring intubation. He was with his family. His advance directives (AD) states not for prolonged life sustaining support, however his wife and daughters plead with the physicians to save his life in order to end the suffering caused by the respiratory distress, and take time to prepare them for a more peaceful death*.*
Open-ended question	Participants were asked to write down what they would do in the same circumstance, taking into consideration the ethical and moral distress faced by ED health care professionals.

### Data analysis

#### Quantitative analysis

Descriptive statistics and variation analysis at different time points were performed on the portfolio data using SAS, version 9.2. The scores of all of the items in a section were added together to obtain a total score for each section.

The analysis of the ethical case score was performed using the ethical template elaborated by Furlan, 2015 [20] (Table 3).

**Table 3:** The ethical template used for ethical case analysis

1. Collecting data and defining the terms used	
medical aspects (current standard, diagnostic and treatment options, benefits and risks), psychological, relational	
2. Definition of the ethical principles at stake and of the various responsibilities	
actors involved in addition to the patient, legal figures, degree of autonomy of the patient and of the persons involved, informed consent and recognition of national and international legal standards	
3. Clarification of conflict of interests and identification of the ethical problems	
ethical problems	
conflicts between principles, team conflicts, conflicts between patient/family and care personnel	
4. Evaluation of possible options	
Analyses of possible courses of action	
5. Justification of choice	
Course of action and ethical principle	
Preferences for one course of action over another	
Resolution of conflict between values	
Principles and facts	

The PI and MP independently noted how many participants identified the steps of the ethical analysis and discussed their findings together to construct the final table. They applied a scoring rubric to each step (*not done* or *fully done*) and compared the responses before and after the training, as described above.

#### Qualitative analysis

Interviews were audio-recorded and transcribed verbatim. Data analysis was conducted by the P. I, together with GA and MP. We performed a theoretically driven thematic analysis [21] by following these analytical stages:

- MP. transcribed the interviews verbatim and shared the transcripts with colleagues. They wrote comments and initial thoughts in a memo;
- MP and GA extracted portions of the text individually and then shared their work to reach an initial agreement. During this stage, they inductively conducted the thematic analysis [21], providing their insights;
- subsequently, they mapped the themes onto the ethics of care framework. Moreover, they focused on the emerging meanings the professionals attributed to their statements, searching for any possible changes in meanings from before to after the training.
- they independently reviewed the themes and allocated portions of the text to the newly reconfigured themes;
- together, they re-defined the themes and re-named them to achieve internal consistency. They also agreed on the final meaning shift.
- GA and MP selected representative extracts from the interviews and drafted the final report, which was checked and amended by all of the authors.

The ethical case’s open-ended questions were qualitatively analysed by GA and MP. Each answer was divided following the ethical template elaborated by Furlan 2015 [20]. They identified significant statements to support quantitative data from a qualitative point of view.

#### Data triangulation

Data triangulation has been performed [22], comparing the qualitative and quantitative data concurrently collected from interviews, ethical skills portfolios and ethical cases.

## Results

The study included 8 participants who were working at an oncological research public hospital “Arcispedale Santa Maria Nuova” of the local health authority of Reggio Emilia. There were three palliative care physicians, two palliative care nurses and three psycho-oncologists. We summarized the participant’s characteristics and their previous training in medical ethics in Table 4**.**

**Table 4:** Participants’ demographic characteristics and previous training in medical ethics

*Code*	*Profession*	*working area*	*Years of work in that area*	*Ethics training BEFORE this course*
1	Psychologist - PhdStudent	Psycho-oncology Unit	trainer	‘No academic ethical course’
2	Physician - Palliative care specialist	Palliative care Unit	7	‘Yes structured theoretical courses, but doubts about the results’
3	Nurse - Palliative care	Palliative care Unit	2	‘Yes short academic, theoretical courses, but I don’t remember anything’
4	Psychologist- Psychotherapist	Psycho-oncology Unit	4	‘No ethics course, but alongside physicians with palliative ethical skills’
5	Nurse - Palliative care	Palliative care Unit	6	‘Never made real training’
6	Physician - Palliative care specialist	Palliative care Unit	6	‘Only some lessons, then coaching a bioethicist in clinical setting’
7	Psychologist- Psychotherapist	Psycho-oncology Unit	3	‘Yes short academic and theorical course’
8	Physician - Palliative care specialist	Palliative care Unit	1,5	‘No academic ethical course’

They all participated in both the pre- and post-training evaluations.

We first present the results separately from the semi-structured interviews, the ethical skills portfolio and the ethical case, and then we ended by integrating the qualitative and quantitative results through data triangulation.

### Semi-structured interviews

The analysis of the interviews led us to identify three overarching themes:

1. Applying ethical reflections in clinical practice
2. Recognizing the ethical problems
3. Being aware of ethical thinking: individual and shared comparisons among the team

These themes emerged with different meanings (defined within the sub-themes) in relation to the pre-training and the post-training data collection.

#### Applying ethical reflections in clinical practice

Ethical reflection in clinical practice shifted from ‘the need to reflect on boundaries themes’ to the ‘need to apply ethical reflection more broadly’.

Before the training programme, participants expected to become more confident in managing such situations arising *‘in a subtle border’* (c.1,13), being able to distinguish *‘what is ethical from what is not’* (c. 6,8) and to implement a ‘*less standard’* approach towards their patients.

*“I would like to get different nuances also in the ethical approach, I mean, I would become so competent to be really able to fit it on my patients” (c. 2, 50).*

After the training programme, participants agreed that the programme ‘*gave (them) a good theoretical framework’* (c. 2.3; 5.1; 6.1) and motivated them to broadly apply *‘reflections and ethical reasoning’* (c. 5.52; 7.3). They also felt more competent (c. 2,28) towards the ethical complexities.

*“I felt more confident with myself in asking questions to patients in order to help me to clarify their problem or dilemma.” (c. 4,30).*

However, according to some of them, the lack of a more applicative practice hinders the transition between the theoretical phase and clinical practice.

*“I feel that I am missing to bring all this down even more in my clinical practice” (c. 7.1).*

#### Recognizing ethical problems

This theme describes participants’ skills in recognizing ethical issues that arise from ‘the simple identification of a list of problems’ to ‘a deeper and more aware ethical approach’, leading them to address ethical conflicts more easily.

Before the training programme, ethical problems were mainly related to communicative aspects of care and to the management of different, sometimes conflicting, visions between patients and their caregivers. The difficulty of emphasizing the patient’s autonomy is also prominently noted.

*“Maybe, it’s the gap between the information the patient has and the real information, because sometimes it seems to me that there is distance, partly because the patient doesn’t ask, partly because the physician doesn’t communicate and this greatly influences the patient’s choices” (c. 3,13).*

After the training programme, most of the participants increased their ability to distinguish their personal and professional sphere from the patient’s autonomy, focusing their attention on providing the most appropriate quality of life for the patient.

*‘Now, I am more inclined to focus on the patient ( … ). Her problem was with herself, with answering the question “what is better for me? Is it better to live less but feel alive?” ( … ) And in that moment I realized. I said “This is an ethical problem, maybe also a dilemma”’ (cod. 4, 33-34).*

#### Being aware of ethical thinking: individual and shared comparisons among the team

The third result highlights the shift of a participant’s attitude towards the decision-making process from ‘sharing the problem among the team’ to ‘individually apply a more conscious and aware ethical thinking’.

Before the training, participants defined the moment of discussion among the rest of the team as *a huge possibility (c. 4.50; 2.45).* Team discussions are essential for the decision-making process.

*“all the decisions I made..(were based on ) consultation with the bioethicist, because we deal with hard care path, there are many implications …* ” (cod. 5,7-8)

After the training programme, some participants referred to a *‘more conscious reflection’,* asking *‘more precise questions’* (c. 2,29) but also better managing the content of the answers, applying the skills acquired during the programme.

*‘It’s like having a tool that you didn’t have before. Before, I stopped to bring up the problem but I couldn’t (manage it)...Then I have all the steps to (understand ) how to deal with it’ (c. 2,35).*

### Ethical skills portfolio

At the end of theoretical part (T1), 7 out of 8 participants reported an increase in their knowledge about the main ethical and legal sources, with a mean relative increase of 65.8% (SD:76.9), while only 4 participants reported an increase in their knowledge about advanced care planning (a mean relative increase of 50.0% (SD:70.8).

A relative increase at T1 in self-reported ability in developing critical thinking (41.9%; SD:37.0), ethical analysis skills (40.1%; SD:24.0), development of ethical behaviours (58.3% ± 48.8) and the ability to conduct ethical consultations (34.5%, SD:26.2) has been observed among all of the participants.

At the end of the ethics consultations (T2), the portfolios showed an overall increase in the development of ethical behaviours (49.0%; SD:30.3) and in the ability to conduct ethical consultations (40.0%; SD:29.4) compared with T0.

Comparing the abilities to develop ethical behaviours and in conducting ethical consultations at T1 and T2, we observed that 33.3 and 42.9% of participants, respectively, reported an increase in their competencies, while 33.3 and 28.6% actually had a slight decrease in their skills.

### Ethical case analysis

All 8 participants performed an ethical case analysis at the beginning (T0) and at the end of the training programme’s theoretical part (T1). The results are presented in the integrating quantitative and qualitative findings.

Analysis of the ethical case scores revealed an increased ability in performing ethical case analysis among the participants, confirming both the development of knowledge and ethical thinking (Table 5**).**

**Table 5:**
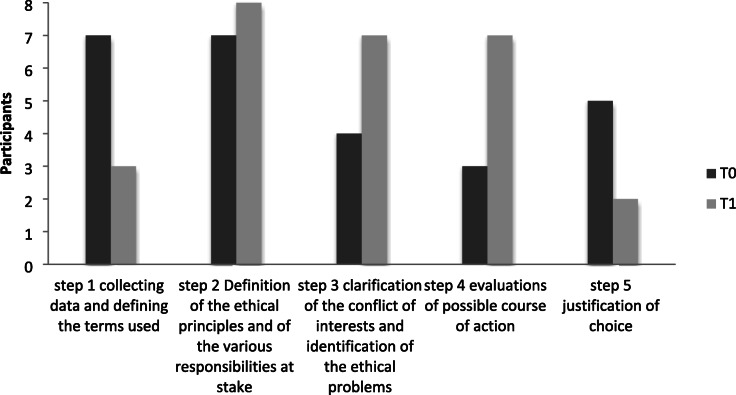
Description of participants’ ethical competences collected from ethical case analysis, before and after the theoretical part

At T1, all 8 participants were able to define ethical principles and the various responsibilities at stake (step 2), confirming their increased theoretical knowledge of medical ethics. Seven of 8 participants also developed abilities in clarifying the conflicts of interests and in identifying the ethical problems (step 3), and in evaluating the possible programme of action (step 4). These findings reveal a decrement in collecting data (step 1), which is probably due to the familiarity with the case. The decrement (from 6 to 2 participants) noted in step 5 and related to the justification of choice, finally confirms both the participants’ difficulty in applying ethical reflections to their practice and overestimation of their knowledge before the programme.

From a qualitative point of view, at T0, participants approached each ethical problem by taking into consideration the patient’s will, as expressed in his AD, and proposing a family meeting to discuss the patient’s care at the end of life, reassuring them that they “*wouldn’t let him go in sufference*” (c.5).

*“I would propose several interviews with physicians, nurses and family, with the aim to share a decision (not only the doctors or only the family), keeping together the clinical data and personal data, likely AD and patient’s value ” (code 3).*

After the training programme, we noted a different approach to the ‘*unconditioned*’ value of a patient’s AD, wondering about the possibility of acting in different way to grasp what truly fit for the patient.

*"The wife’s speeches would develop an inner ethical dilemma in me. It would be generated by the doubt that the patient may suggested change of opinion and that his wife got it" (c.4).*

#### Data’s triangulation

The participants were very active and participatory throughout the training programme and research: they all attended the course from the beginning to the end; they participated at the evaluation at T0,T1,T2; moreover, ethics consultation activity is based on daily case reports from participants. The triangulation of the data led, in most cases, to both confirmatory and novel results. The results converge towards the participants’ increased knowledge and ethical skills, performance and competences. In both quantitative and qualitative research, the results, in synthesis, highlight:

1. (Knowledge and skills): participants developed their ethical knowledge, and a deeper ethical awareness. The increase in ethical knowledge and skills led them to better understand the role of ethics in their clinical practice. (Theme 3, portfolio, ethical case)
2. (Competences): the participants improved their competences in recognizing the ethical problem. They felt more confident and motivated to widely apply “ethical reflections and reasonings in their daily practice.” (Theme 1–2, portfolio)
3. (Change in practical performances): Participants approached ethical problems differently in their clinical practice, both improving their individual capacity to apply ethical thinking in their practice and in searching for more personalized care. (Portfolio, ethical case)

## Discussion

The present work describes the piloting of a new specialized training programme in medical ethics dedicated to a hospital-based PCU along with its evaluation. The study was aimed at evaluating, both quantitatively and qualitatively, the training’s impact on trainees concerning their increased ethical skills.

A subjective evaluation was preferred due to the following training’s characteristics: first of all, this was a CME, so we need flexible tools to highlights healthcare professionals’opinions and experiences; secondly, the course was designed for experienced healthcare professionals with different training needs. For this reason, a self-reported approach is crucial to better organize and evaluate the training.

Our results showed a change in participant’s practical performance, concerning the development of an individual capacity to address ethical issues in a clinical setting and further personalization of the care, in order to provide a more appropriate quality of life. Team discussions of complex situations remained a fundamental aspect of ethics performance, as well as the recommendations on specific issues [23, 24] despite the increment of individual skills. Team discussions made the professionals feel more confident and less concerned about the continuous ethical challenges [25] and more capable of meeting the needs of the patient and the family [26, 27].

The study design starts from an accurate literature review while collecting the participants’ specific needs during pre-training interviews. The pre-training interviews were helpful in identifying participants’ needs and expectations, but we considered it necessary to conduct a systematic review of the literature to better understand the outcomes of ethics educational programmes in palliative care.

We integrated theoretical-interactive lessons [12, 28], workshops based on daily clinical practice cases [9, 29–31] and ethics consultation activity [5] with the aim to facilitate the transition from theory to practice. Nevertheless, results highlighted a lack of a more applicative practice.

We trained a multidisciplinary palliative care team, made up of physicians, nurses and psycho-oncologists who worked together and this represents a novel aspect, compared to most of the literature [8–11]. Taking care of a patient’s needs requires a multidisciplinary team: consequently, the team’s members should be trained together and not in different programmes related to their profession [5, 12, 13]. Despite this, we have to take into account that this is a small multidisciplinary team is used to working together: future studies will have to demonstrate how it can be implemented with a larger group.

We implemented a pre-post evaluation [32, 33] of the training after adopting a specific methodology, which led us to describe the development of new knowledge, new awareness and changes in both performance and competence [34]. On the contrary, most of the training programmes described by literature have been evaluated only in terms of participants’ perception and satisfaction [5, 12, 13].

Increasing ethical awareness represents an important achievement of our training: as underlined by Milken and Grace, increasing ethical awareness helps health care professionals apply ethical decision-making, recognizing the unique interests and wishes of individuals, in line with an ethic of care [35, 36].

Our programme incremented also participants’ ethical competence. According to Guevara-Lòpez [37], ethical competencies are central elements for the development of palliative care, especially concerning truth-telling, which implies bidirectional trust between patients and healthcare providers [38].

The study bears several risk of selection bias because of the characteristics of the study participants: hospital-based PCU and BU who had been working together for 3 years before this training programme, so the health care professionals involved were particularly sensitive to ethical issues in clinical practice. Moreover, the small sample size represents a strong limitation for quantitative analysis.

Lastly, this study was conducted in Italy and further research needs to be conducted in other settings to assess the transferability of the findings by a cultural point of view.

## Conclusion

The participants appreciated the innovative structure of the training, especially regarding the integration of the theoretical-interactive and practical parts. However, they recommended increasing the number of concrete occasions for ethical supervision and practical application of what they learned during the programme.

The implementation and evaluation of our T4EPC training programme has some practical implications that need to be analysed and evaluated. First of all, the development of advanced ethical skills within a hospital-based PC team may improve the quality of life of the patients and their families. In addition, health care professionals with advanced ethical competencies are able to educate patients and their families towards more active participation in the decision-making process. We can argue that the first group of professionals who completed this training can actively collaborate with the Bioethics Unit to support other non-trained professionals in the difficult ethical choices they encounter in their daily activities.

## Data Availability

All data generated or analysed during this study are included in this published article (and its supplementary information files).

## Appendix

**Table 6:** Semi-structured pre and post training interview’s guide

Thematic Area	Topic	Pre-training interviews’ Illustrative questions	Post-training interviews’ Illustrative questions post
Introduction		-Would you like to describe your previous experiences regarding training courses on medical ethics?	
Participant’s expectations	Participants’ expectations, desires and ideas related to the training programme.	- What are your expectations and wishes regarding the ethics training?	-Thinking about the ethics training you attended, do you think your expectations have been satisfied?
		-What do you expect from an ethics training for palliative care professionals?	
			-What about your whishes?
			-Did you change some of your previous ideas about the course?
Ethical issues of participants’ clinical practice	Ethical problems experienced by participants in their clinical practice as critical issues and / or difficulties.	- What are the main ethical issues you usually experience in your daily clinical practice?	- What are the main ethical issues you usually experience in your daily clinical practice?
		-Can you give an example?	- Can you give an example?
		-Can you explain the difficulties you face?	-Can you explain the difficulties you face?
Professional needs	Participants’ background regarding ethical issues in clinical practice.	-Based on your experience, how did you behave in such circumstances?	-Based on your experience, how did you behave in such circumstances?
		-Did you ask for help or collaboration with someone?	-Did you ask for help or collaboration with someone?
		-How did you experience the possible solution?	-How did you experience the possible solution?
Conclusion		-Are there any important things you would like to tell us?	-Are there any important things you would like to tell us?
		- Would you like to share something else?	-Would you like to share something else?

## Abbreviations

PCU: Palliative Care Unit
BU: Bioethics Unit
T4EPC: Teach for Ethics in Palliative Care
